# Supplementary material for: Enabling direct-growth route for highly efficient ethanol upgrading to long-chain alcohols in aqueous phase
Source: Nat Commun. 2023 Dec 1;14:7935. doi: 10.1038/s41467-023-43773-3 (PMC10692112; doi:10.1038/s41467-023-43773-3)
Supplement: Supplementary file 1 — Supplementary Information [file 41467_2023_43773_MOESM1_ESM.pdf]

# Supplementary Information

# Enabling direct-growth route for highly efficient ethanol upgrading to long-chain alcohols in aqueous phase

Juwen Gu[1,6], Wanbing Gong[2,6], Qian Zhang[1,3,4,6], Ran Long[2], Jun Ma[2], Xinyu Wang[2], Jiawei Li[2], Jiayi Li[2], Yujian Fan[1], Xinqi Zheng[1], Songbai Qiu [1,3,4]*, Tiejun Wang[1,3,4]* and Yujie Xiong[2,5]*

[1]School of Chemical Engineering and Light Industry, Guangdong University of Technology, Guangzhou 510006, China.

[2]Hefei National Research Center for Physical Sciences at the Microscale, Collaborative Innovative Center of Chemistry for Energy Materials (iChEM), School of Chemistry and Materials Science, National Synchrotron Radiation Laboratory, School of Nuclear Science and Technology, University of Science and Technology of China, Hefei, Anhui 230026, China.

[3]Guangdong Provincial Key Laboratory of Plant Resources Biorefinery, Guangzhou 510006, China.

[4]Guangzhou Key Laboratory of Clean Transportation Energy and Chemistry, Guangzhou 510006, China.

[5]Suzhou Institute for Advanced Research, Nano Science and Technology Institute, University of Science and Technology of China, Suzhou, 215123, China.

[6]These authors contributed equally: Juwen Gu, Wanbing Gong, Qian Zhang.

* Corresponding author.

Tel: 0086-0551-63606657, E-mail: qiusb@gdut.edu.cn, tjwang@gdut.edu.cn, yjxiong@ustc.edu.cn

# Table of Contents

**1. Supplementary synthesis of catalysts**

**1)** Chemicals

**2)** Synthesis of the NiSn@C catalyst

**3)** Synthesis of the Ni@NC catalyst

**2. Supplementary catalytic experiments**

**1)** 2-Butenal hydrogenation experiments

**2)** *n*-Butanal condensation experiments

**3)** Product analysis

**4)** Surface sulfur coverage and Ni active sites quantification

**3. Supplementary Figures**

**Supplementary Fig. 1** | Schematic illustration of the preparation procedure for Ni@C-$S_x$ catalysts.

**Supplementary Fig. 2** | XRD patterns of typical Ni@C-$S_x$ catalysts.

**Supplementary Fig. 3** | SEM images of the (**a**, **b**) Ni@C-$S_0$ and (**c**, **d**) Ni@C-$S_{1/30}$ catalysts.

**Supplementary Fig. 4** | (**a**) TEM image, (**b**) HRTEM image, (**c**) HAADF-STEM image and (**d-g**) EDS elemental mappings of the Ni@C-$S_{1/30}$ catalyst.

**Supplementary Fig. 5** | (**a**, **b**) TEM images and (**c**, **d**) HRTEM images of the Ni@C-$S_0$ catalyst.

**Supplementary Fig. 6** | TEM images of the Ni@C-$S_{1/30}$ catalyst after treated by 1.0 M HCl.

**Supplementary Fig. 7** | (**a**) XPS survey spectra. High-resolution (**b**) Ni $2p_{3/2}$, (**c**) S 2p and (**d**) C 1s XPS spectra for typical Ni@C-$S_x$ catalysts.

**Supplementary Fig. 8** | *In situ* DRIFTS for CO adsorption over the (**a**) Ni@C-$S_0$, (**b**) Ni@C-$S_{1/30}$ and (**c**) Ni@C-$S_{1/25}$ catalysts.

**Supplementary Fig. 9** | (**a**) XPS survey spectra. High-resolution (**b**) Ni $2p_{3/2}$, (**c**) S 2p and (**d**) C 1s XPS spectra of the Ni@C-$S_{1/30}$ catalyst for different $Ar^+$ etching times.

**Supplementary Fig. 10** | Detailed alcohol distribution, step-growth plot and $\alpha$ value over Ni@C-$S_x$ catalysts with different S/Ni molar ratios: (**a**) 0, (**b**) 1/60, (**c**) 1/30, (**d**) 1/25, (**e**) 1/20.

**Supplementary Fig. 11** | GC profiles of liquid products obtained from reaction at 180 °C for 12 h over the Ni@C-$S_{1/30}$ catalyst.

**Supplementary Fig. 12** | The corresponding molecular structures of the products in Supplementary Fig. 11 and Supplementary Fig. 14.

**Supplementary Fig. 13** | The performance of Ni@C-$S_{1/30}$ catalyst under different reaction conditions. (**a**) EtOH conversion and product selectivity for various reaction times at 180 °C. (**b**) EtOH conversion and product selectivity at various temperatures within 12 h. **Supplementary Fig. 14** | GC profiles of liquid products obtained from a two-stage intensification process reaction (180 °C for 6 h and then heating to 250 °C for another 6 h) over the Ni@C-$S_{1/30}$ catalyst.

**Supplementary Fig. 15** | XRD patterns of the Ni@C-$S_{1/30}$ catalyst before and after six reaction cycles.

**Supplementary Fig. 16** | (**a**) XPS survey spectra. High-resolution (**b**) Ni $2p_{3/2}$, (**c**) S 2p and (**d**) C 1s XPS spectra for the Ni@C-$S_{1/30}$ catalyst before and after six reaction cycles.

**Supplementary Fig. 17** | *n*-Butanal-TPD/MS profiles over the NiSn@C and Ni@NC catalysts: (**a**) Signals of *n*-butanal (*m/z* = 72) and (**b**) signals of 1-propane (*m/z* = 44).

**Supplementary Fig. 18** | Temperature-dependent *in situ* DRIFTS spectra in the range of 1600−1900 $cm^{-1}$ for *n*-butanal adsorption on the (**a**) Ni@C-$S_0$, (**b**) Ni@C-$S_{1/30}$ and (**c**) Ni@C-$S_{1/25}$ catalysts.

**Supplementary Fig. 19** | Catalyst models of Ni (111) with different sulfur coverage: (**a**) 0%, (**b**) 3%, (**c**) 14%, (**d**) 20%, (**e**) 25%, (**f**) 36%, (**g**) 56%.

**Supplementary Fig. 20** | The stable adsorption configurations of *n*-butanal molecule on Ni (111) with different sulfur coverage: (**a**) 0%, (**b**) 3%, (**c**) 14%, (**d**) 20%, (**e**) 25%, (**f**) 36%, (**g**) 56%.

**Supplementary Fig. 21** | Configurations of $CH_3CH_2OH$* dehydrogenation to $CH_3CH_2O$* on Ni (111) with different sulfur coverage: (**a-c**) 0%, (**d-f**) 14%, (**g-i**) 25%.

**Supplementary Fig. 22** | Configurations of intermediate $CH_3CO$* decomposition to $CH_3$* and CO* on Ni (111) with different sulfur coverage: (**a-c**) 0%, (**d-f**) 14%, (**g-i**) 25%.

**Supplementary Fig. 23** | Detailed alcohol distribution, step-growth plot and *α* value as a function of DMSO fraction in aqueous EtOH over the Ni@C-$S_0$ catalyst: (**a**) 0, (**b**) 0.006, (**c**) 0.009, (**d**) 0.012 (**e**) 0.024.

**Supplementary Fig. 24** | XRD patterns of the Ni@C-$S_0$ catalyst before and after reaction in aqueous EtOH solution with 0.006% DMSO fraction.

**Supplementary Fig. 25** | (**a**) XPS survey spectra. High-resolution (**b**) Ni $2p_{3/2}$, (**c**) S 2p and (**d**) C 1s XPS spectra for the Ni@C-$S_0$ catalyst before and after reaction in aqueous EtOH solution with 0.006% DMSO fraction.

**Supplementary Fig. 26** | (**a**) TEM image, (**b**) HRTEM image, (**c**) HAADF-STEM image and (**d-g**) EDS elemental mappings of the Ni@C-$DMSO_{0.006}$ catalyst.

**Supplementary Fig. 27** | Detailed alcohol distribution, step-growth plot and *α* value

over Ni@C-$S_{1/30}$-y catalysts prepared with other sulfur precursors: (**a**) $NiSO_4$, (**b**) LA and (**c**) SDS.

**Supplementary Fig. 28** | Detailed alcohol distribution, step-growth plot and *α* value over different catalysts under optimized conditions: (**a**) Ni@C-$S_0$, (**b**) Ni@C-$DMSO_{0.006}$, (**c**) Ni@C-$S_{1/30}$, (**d**) Ni@C-$S_{1/30}$-$NiSO_4$, (**e**) Ni@C-$S_{1/30}$-LA and (**f**) Ni@C-$S_{1/30}$-SDS.

## 4. Supplementary Tables

**Supplementary Table 1** | Chemical compositions and textural properties of typical Ni@C-$S_x$ catalysts.

**Supplementary Table 2** | Catalytic performance of aqueous EtOH coupling over Ni@C-$S_x$ catalysts with different S/Ni molar ratios.

**Supplementary Table 3** | EtOH conversion and product selectivity as a function of reaction time over the Ni@C-$S_{1/30}$ catalyst.

**Supplementary Table 4** | Effect of reaction temperature on the catalytic performance of Ni@C-$S_{1/30}$ catalyst.

**Supplementary Table 5** | Effect of two-stage heating program with different second step temperature on the catalytic performance of Ni@C-$S_{1/30}$ catalyst.

**Supplementary Table 6** | Catalytic performance comparison among the Ni@C-$S_{1/30}$ catalyst and other previously reported catalysts.

**Supplementary Table 7** | Stability of the Ni@C-$S_{1/30}$ catalyst.

**Supplementary Table 8** | Catalytic performance of aqueous EtOH coupling over typical Ni@C-$S_x$ catalysts with different S/Ni molar ratios.

**Supplementary Table 9** | Catalytic performance of Ni@C-$S_x$ catalysts for 2-butenal hydrogenation.

**Supplementary Table 10** | Dehydrogenation and hydrogenation rate of Ni@C-$S_x$ catalysts.

**Supplementary Table 11** | Aldol condensation of *n*-butanal catalyzed by NaOH.

**Supplementary Table 12** | Surface sulfur coverage and Ni active sites of typical Ni@C-$S_x$ catalysts.

**Supplementary Table 13** | Catalytic performance of aqueous EtOH coupling over Ni@C-$DMSO_z$ catalysts with different DMSO fractions in aqueous EtOH.

**Supplementary Table 14** | Catalytic performance of aqueous EtOH coupling over unmodified Ni@C-$S_0$ and Ni@C-$S_{1/30}$-y catalysts prepared with different sulfur precursors.

**Supplementary Table 15** | The catalytic performance of typical catalysts under the optimized two-stage intensification process.

## 5. Reference

## 1. Supplementary synthesis of catalysts

**1) Chemicals.** Nickel (II) nitrate hexahydrate ($Ni(NO_3)_2·6H_2O$, 98.0%) and acetone (99.5%) were purchased from Guangzhou Chemical Reagent Factory Co., Ltd., China. Citric acid monohydrate (CAM, 99.5%), L-cysteine (L-Cys, 99.0%), nickel (II) acetate tetrahydrate ($Ni(AcO)_2·4H_2O$, 99.9% metals basis), nickel(II) sulfate ($NiSO_4$, 99.0%), tin (IV) chloride pentahydrate ($SnCl_4·5H_2O$, 99.0%), lipoic acid (LA, 99%), sodium dodecyl sulfate (SDS, 99.0%), sodium hydroxide (NaOH, 97%), 1-pentanol (99.5%), methanol (MeOH, 99.5%) and ethanol (EtOH, 99.7%) were purchased from Macklin Biochemical Co., Ltd., China. 2-Butenal (98.0%) and *n*-butanal (98.0%) were purchased from Shanghai Xianding Biotechnology Co., Ltd., China. Nonionic polyacrylamide (PAM, with molecular weight between 200 and 1400 million) was purchased from Shanghai Aladdin Biochemical Technology Co., Ltd., China. All of the chemicals were obtained from commercial sources and used without further purification.

**2) Synthesis of the NiSn@C catalyst.** The NiSn@C catalyst was prepared according to literature[1]. CAM (25.4 mmol) was dissolved in 15.0 mL of deionized water and stirred until completely dissolved. $Ni(NO_3)_2·6H_2O$ (12.7 mmol) and $SnCl_4·5H_2O$ (0.6 mmol) were added into the above solution in sequence with string. After vigorously stirring at room temperature for 2 h, the mixture was gradually gelled by evaporation at 100 °C and then dried in an oven at 100 °C for 48 h. The fully dried solid gels were then annealed at 600 °C for 4 h at a heating rate of 5 °C $min^{-1}$ under $N_2$ atmosphere.

**3) Synthesis of the Ni@NC catalyst.** The Ni@NC catalyst was prepared according to literature[2]. $Ni(AcO)_2·4H_2O$ (17.6 mmol) and PAM (35.2 mmol) were dissolved in 150.0 mL of deionized water, followed by vigorous stirring at room temperature to generate a homogeneous hydrogel. The hydrogel was then evaporated at 85 °C until fully dried. After that, the dried gel was carbonized at 500 °C for 2 h at a heating rate of 10 °C $min^{-1}$ under $N_2$ atmosphere.

## 2. Supplementary catalytic experiments

**1) 2-Butenal hydrogenation experiments.** Typically, Ni@C-$S_x$ catalyst (0.05 g), 50.0 wt% EtOH (40.0 mL) and 2-butenal (14.0 mmol) were placed into a 70 mL reactor. The inner atmosphere was then repeatedly evacuated and recharged five times with $H_2$ (0.1 MPa) and then filled with pure $H_2$ (2 MPa). Finally, the reactor was heated to 180 °C and held for 1 h. After the reaction finished, the reactor was immediately cooled to room temperature with a water bath.

**2) *n*-Butanal condensation experiments.** The *n*-butanal condensation experiment was carried out at room temperature. *n*-butanal (69.4 mmol) was dissolved in 50.0 wt% EtOH solution (20.0 mL) in a 100 mL beaker. Then another 50.0 wt% EtOH solution (20.0 mL) containing 2.5 mmol NaOH was poured into the 100 mL beaker, followed by vigorous stirring at room temperature for 10 min. Finally, a separation funnel was used to rapidly separate the oil and aqueous phase products.

**3) Product analysis.** After the reaction, a portion of the gaseous products (100 mL) was collected in a gas bag, and the volume of remaining gaseous products was then measured by the drainage method. The gaseous products were analyzed by gas chromatograph (Agilent 8860 GC) equipped with a thermal conductivity detector (TCD) and a flame ionization detector (FID).

The liquid products were identified by gas chromatography–mass spectrometry (GC–MS, Shimadzu QP2014) system with an RTX-WAX column. The liquid products were quantitatively analyzed with an internal standard method by GC system (Shimadzu 2010 Pro) with an HP-INNOWax column. 1-pentanol was used as the internal standard. Methanol and acetone were used as solvents for the aqueous and oil phase products, respectively. The carbon balance, conversion and selectivity were evaluated by using the following equations:

$$\text{Carbon balance (C-mol\%)} = \frac{\sum Moles\ of\ carbon\ in\ all\ detected\ products}{Moles\ of\ carbon\ in\ feedstock} \times 100\% \quad (1)$$

$$\text{Conversion (C-mol\%)} = (1 - \frac{Moles\ of\ feedstock\ after\ the\ reaction}{Moles\ of\ feedstock\ before\ the\ reaction}) \times 100\% \quad (2)$$

$$\text{Selectivity (C-mol\%)} = \frac{Moles\ of\ carbon\ in\ the\ target\ product}{Moles\ of\ carbon\ in\ all\ product} \times 100\% \quad (3)$$

The chain growth probability ($\alpha$) was calculated according to step-growth model:

$$\ln\left(\frac{W_n}{n}\right) = n\ln\alpha + \frac{\ln(1-\alpha)^2}{\alpha} \quad (4)$$

where n is the number of monomer (EtOH) in chain, $W_n$ is the mass fraction of the alcohols with a monomer number of $n$, and $\alpha$ is chain growth probability. Plotting ln ($W_n/n$) *versus* n, the chain

growth probability ($\alpha$) can be obtained by calculating the slope ($\ln\alpha$).

The reaction rates were evaluated by using the following equations:

$$\text{EtOH dehydrogenation rate } (\text{mmol}\cdot g_{cat}^{-1}\cdot h^{-1}) = \frac{\textit{Moles of reacted EtOH} \times 2}{\textit{Catalyst mass} \times \textit{Reaction time}} \quad (5)$$

$$\text{Hydrogenation rate } (\text{mmol}\cdot g_{cat}^{-1}\cdot h^{-1}) = \frac{\textit{Moles of n-butanal} \times 2 + \textit{Moles of n-butanol} \times 4}{\textit{Catalyst mass} \times \textit{Reaction time}} \quad (6)$$

$$\text{C-C bond formation rate } (\text{mmol}\cdot g_{cat}^{-1}\cdot h^{-1}) = \frac{\textit{Moles of reacted n-Butanal}}{2 \times \textit{Catalyst mass} \times \textit{Reaction time}} \quad (7)$$

**4) Surface sulfur coverage and Ni active sites quantification:** The Ni active sites were quantified via $N_2O$ titration using the pulse technique on a Micromeritics AutoChem II. Catalyst samples (20 mg) were reduced at 450 °C for 1 h (10 °C·min$^{-1}$) under a flow of 10 % $H_2$/Ar, and then switched to Ar flow and cooled to 90 °C. Finally, the pulsed $N_2O$ titration was carried out at 90 °C.

The active sites on the S-free Ni@C-$S_0$ catalyst were calculated based on the assumption of $N_2O_{consumed}/Ni_{surface}$ = 1/1. Each Ni atom on the surface of S-free Ni@C-$S_0$ catalyst can become the active site for *n*-butanal adsorption. The quantity of Ni active sites on S-doped Ni@C-$S_x$ catalysts was calculated by assuming that the number of surface Ni atoms is the same as those of S-free Ni@C-$S_0$ and S-doped Ni@C-$S_x$ catalysts, each Ni atom and S atom on the surface can be oxidized by one $N_2O$ molecule, while one S atom occupies three surface Ni atoms (the Ni atoms occupied by S atoms cannot serve as active sites for *n*-butanal adsorption). The surface sulfur coverage and Ni active sites on S-doped Ni@C-$S_x$ catalysts can be calculated as the following formula:

$$\text{Surface sulfur coverage (\%)} = \frac{n_{Ni@C\text{-}S_x} - n_{Ni@C\text{-}S_0}}{n_{Ni@C\text{-}S_0}} \times 100\% \quad (8)$$

$$\text{Surface Ni active sites } (\mu\text{mol}\cdot g^{-1}) = n_{Ni@C\text{-}S_0} - (n_{Ni@C\text{-}S_x} - n_{Ni@C\text{-}S_0}) \times 3 \quad (9)$$

in which the $n_{Ni@C\text{-}S_x}$ and $n_{Ni@C\text{-}S_0}$ were referred to as the mole of consumed $N_2O$ over per gram of S-doped Ni@C-$S_x$ and S-free Ni@C-$S_0$ catalysts, respectively.

## 3. Supplementary Figures

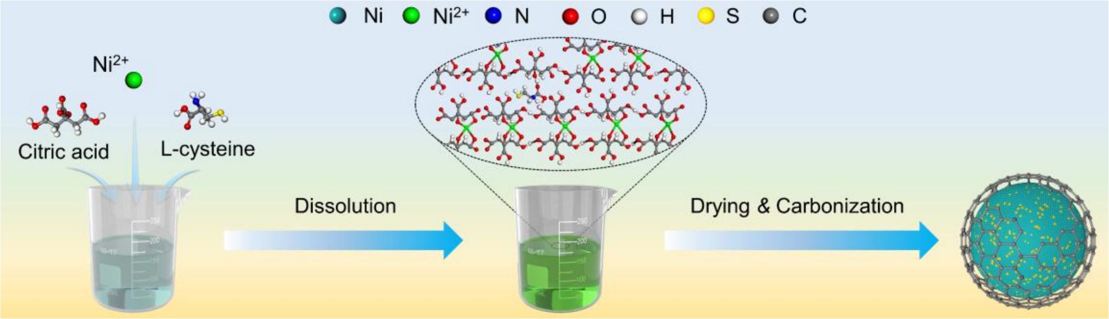


**Supplementary Fig. 1** | Schematic illustration of the preparation procedure for Ni@C-$S_x$ catalysts.

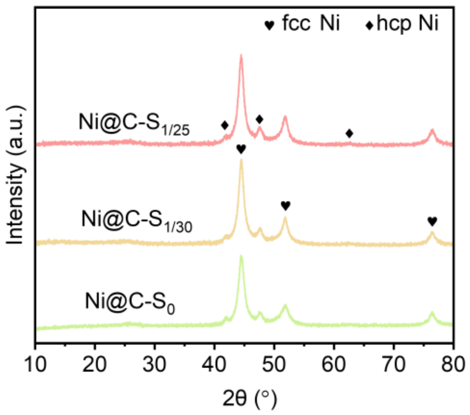

**Supplementary Fig. 2** | XRD patterns of typical Ni@C-$S_x$ catalysts.

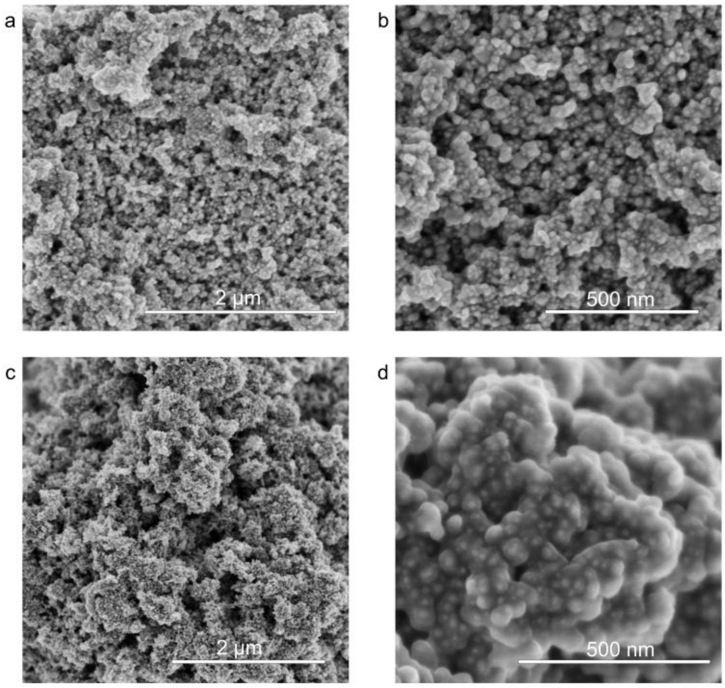


**Supplementary Fig. 3** | SEM images of the (**a**, **b**) Ni@C-$S_0$ and (**c**, **d**) Ni@C-$S_{1/30}$ catalysts.

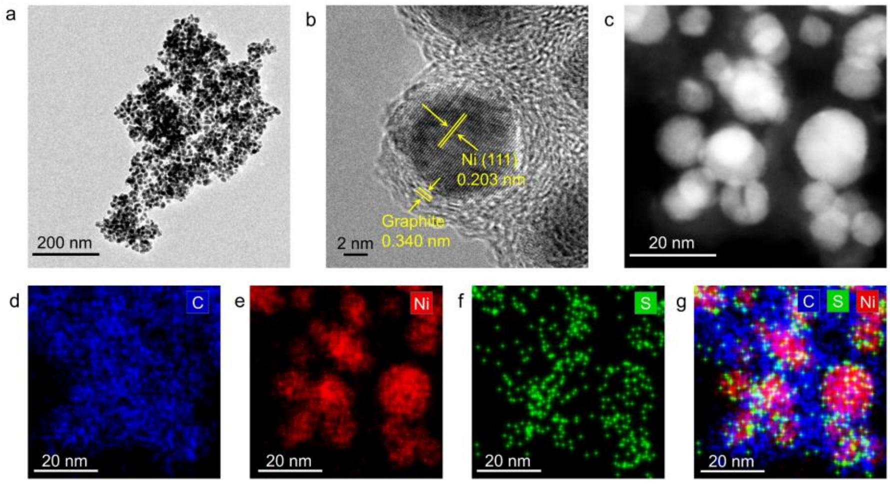


**Supplementary Fig. 4** | (**a**) TEM image, (**b**) HRTEM image, (**c**) HAADF-STEM image and (**d-g**) EDS elemental mappings of the Ni@C-$S_{1/30}$ catalyst.

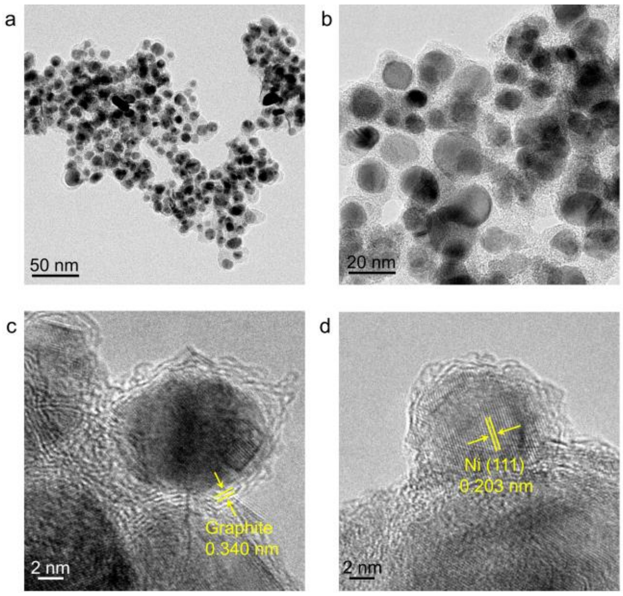


**Supplementary Fig. 5 |** (**a**, **b**) TEM images and (**c**, **d**) HRTEM images of the Ni@C-$S_0$ catalyst.

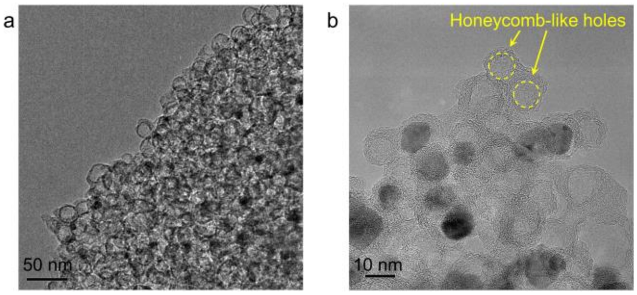


**Supplementary Fig. 6** | TEM images of the Ni@C-$S_{1/30}$ catalyst after treated by 1.0 M HCl.

To visually showcase the abundance of exposed active Ni sites on the catalyst, the Ni@C-$S_{1/30}$ catalyst was introduced to an excess hydrochloric acid solution (1.0 M HCl) and kept standing for 30 min. According to the TEM images of Ni@C-$S_{1/30}$ catalyst after acid treatment (Supplementary Fig. 6), only a small number of Ni NPs was tightly wrapped by the graphitized carbon layers, whereas the majority of the exposed Ni NPs were etched and completely eliminated by 1.0 M HCl. As a result, numerous honeycomb-like holes were remained on the carbon layers.

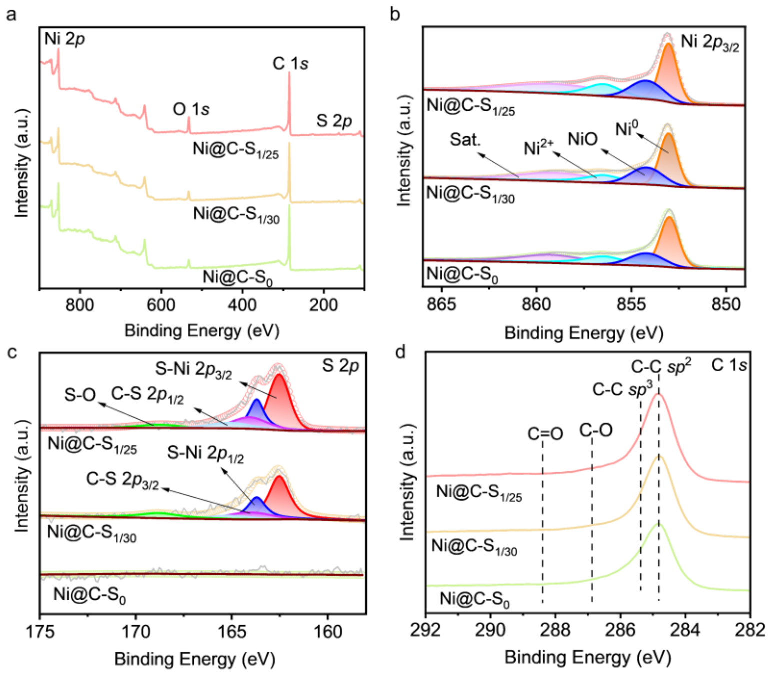


**Supplementary Fig. 7** | (**a**) XPS survey spectra. High-resolution (**b**) Ni 2$p_{3/2}$, (**c**) S 2*p* and (**d**) C 1*s* XPS spectra for typical Ni@C-$S_x$ catalysts.

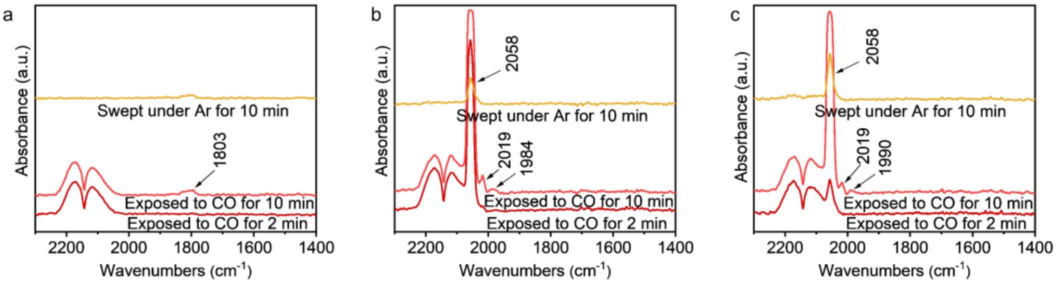


**Supplementary Fig. 8** | *In situ* DRIFTS for CO adsorption over the (**a**) Ni@C-$S_0$, (**b**) Ni@C-$S_{1/30}$ and (**c**) Ni@C-$S_{1/25}$ catalysts.

As CO molecules tend to form linear adsorption and produce $Ni(CO)_4$ species on the S-doped Ni surface[3-6], we employed *in situ* DRIFTS to study CO chemisorption on typical Ni@C-$S_x$ catalysts. As seen in Supplementary Fig. 7, the peaks at 2120 and 2170 $cm^{-1}$ represent CO gas molecules. The bands at 1803 and 1984/1990 $cm^{-1}$ are observed, which are consistent with CO adsorbed on the threefold hollow and bridge sites, respectively[7, 8]. In addition, the bands at 2019 and 2058 $cm^{-1}$ can be assigned to linearly adsorbed CO[9] and $Ni(CO)_4$[10, 11], respectively. For the Ni@C-$S_0$ catalyst, only threefold hollow adsorption of CO can be observed and the chemisorbed CO species on Ni sites are still stable after swept by Ar flow. Distinctly, linear adsorption of CO and $Ni(CO)_4$ both emerge on the Ni@C-$S_{1/30}$ and Ni@C-$S_{1/25}$ catalysts, and all bands decrease in intensity after being swept by Ar flow. Meanwhile, the weak band at 1984 $cm^{-1}$ shifts to 1990 $cm^{-1}$ as the S/Ni ratio rises up from 1/30 to 1/25, indicating that the bridge adsorption of CO is further weakened. Taken together, the *in situ* DRIFTS spectra clearly confirm the presence of sulfur covering the surface of Ni with a lower affinity for CO in S-doped Ni@C-$S_x$ catalysts.

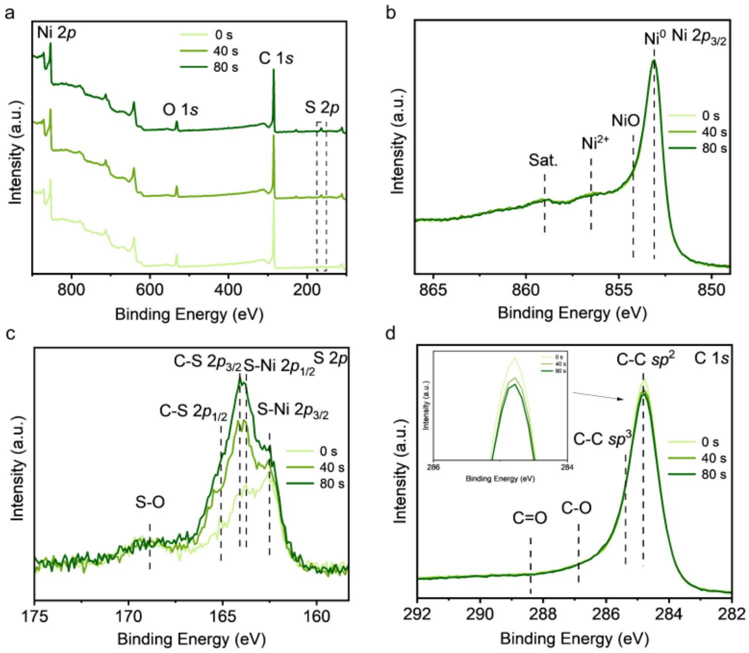


**Supplementary Fig. 9** | (**a**) XPS survey spectra. High-resolution (**b**) Ni 2$p_{3/2}$, (**c**) S 2$p$ and (**d**) C 1$s$ XPS spectra of the Ni@C-$S_{1/30}$ catalyst for different $Ar^+$ etching times.

The Ni@C-$S_{1/30}$ catalyst was etched by $Ar^+$ beam for different time to reveal subsurface information. The intensity of the C1$s$ attenuates as a function of the etching time, indicating that the carbon on the surface of catalyst was gradually removed by $Ar^+$ beam. On the contrary, the S 2$p$ spectra show that the intensity of the peaks corresponding to Ni–S and C–S species increases significantly by prolonging the etching time, suggesting that the sulfur species at the interface of the Ni surface and the adjacent carbon layer start to emerge after removing the surface carbon layer.

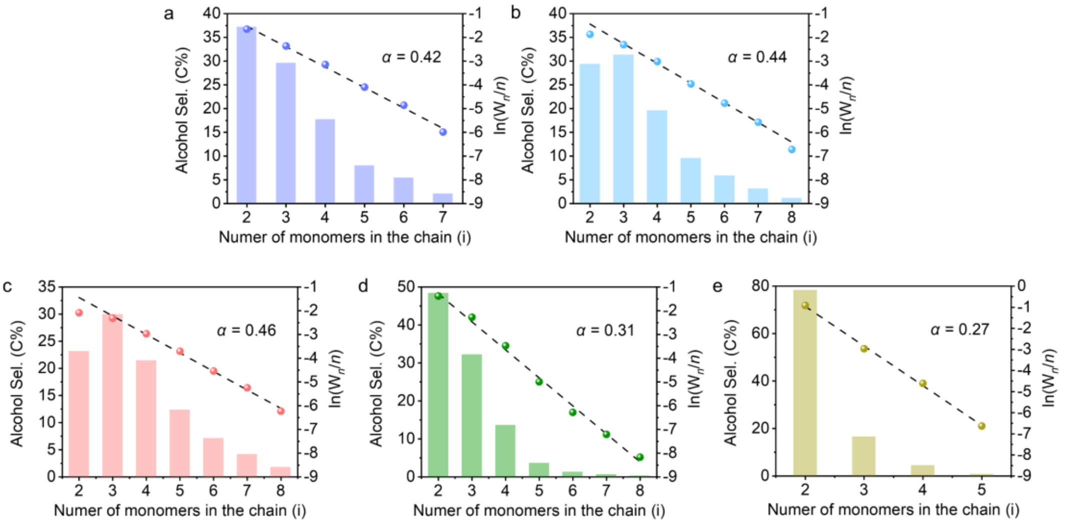


**Supplementary Fig. 10** | Detailed alcohol distribution, step-growth plots and $\alpha$ values over Ni@C-$S_x$ catalysts with different S/Ni molar ratios: (**a**) 0, (**b**) 1/60, (**c**) 1/30, (**d**) 1/25, (**e**) 1/20.

The chain growth probabilities ($\alpha$) were obtained by fitting the results obtained for the alcohol products using step-growth model. Supplementary Fig. 9 shows the variation in the probability of chain growth for alcohol products as a function of S/Ni molar ratios. This $\alpha$ increases from 0.42 to 0.46 as the S/Ni molar ratio increases from 0 to 1/30, indicating that the introduction of appropriate sulfur can significantly increase the selectivity of LAS. Notably, in addition to the increase of $\alpha$, the $\ln(W_n/n)$ values of butanol are gradually lower than the predicted step-growth plots, implying that the LAS selectivity over Ni@C-$S_x$ catalysts has exceeded the value predicted by the step-growth model.

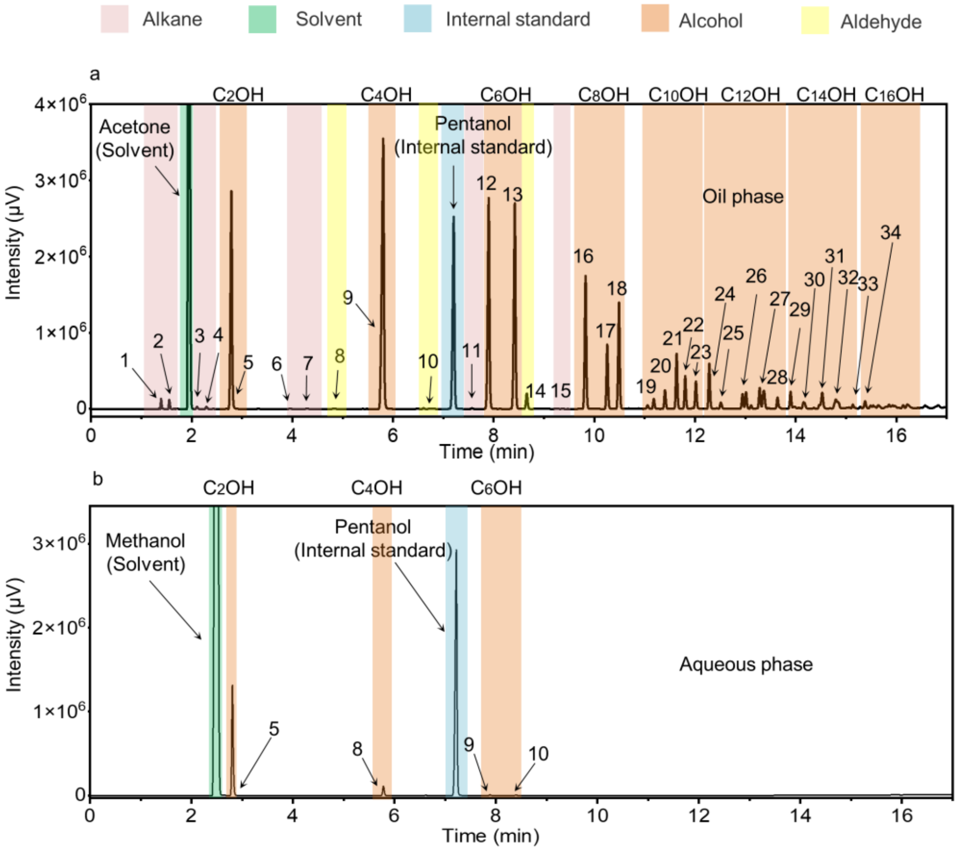


**Supplementary Fig. 11** | GC profiles of liquid products obtained from reaction at 180 °C for 12 h over the Ni@C-$S_{1/30}$ catalyst.

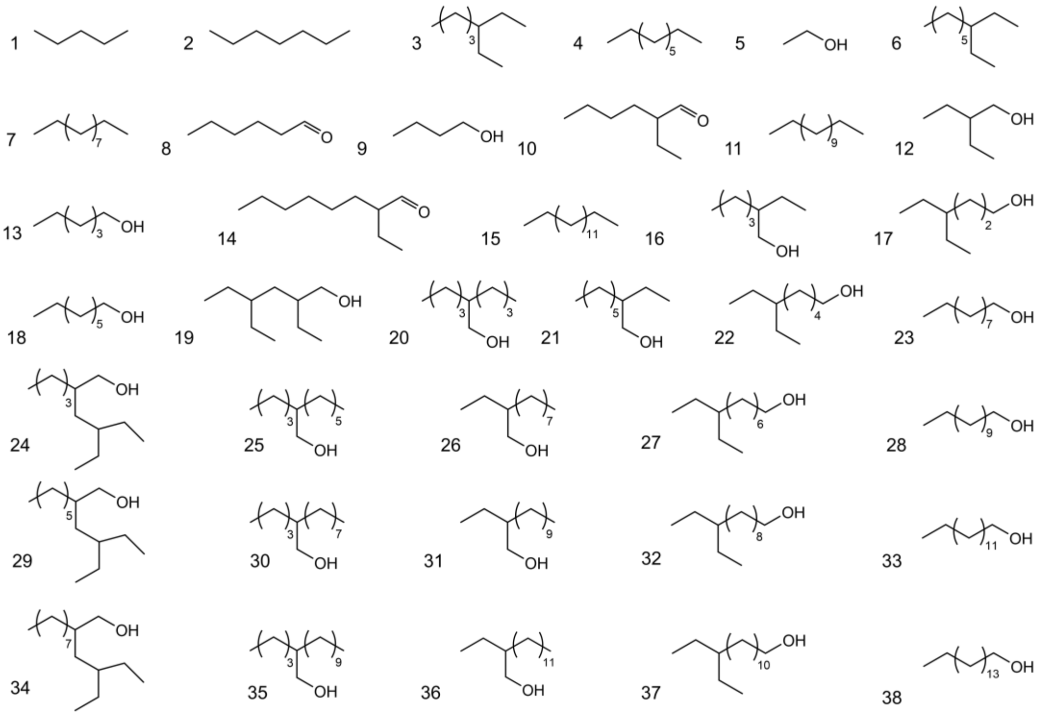

**Supplementary Fig. 12 |** The corresponding molecular structures of the products in Supplementary Fig. 11 and Supplementary Fig. 14.

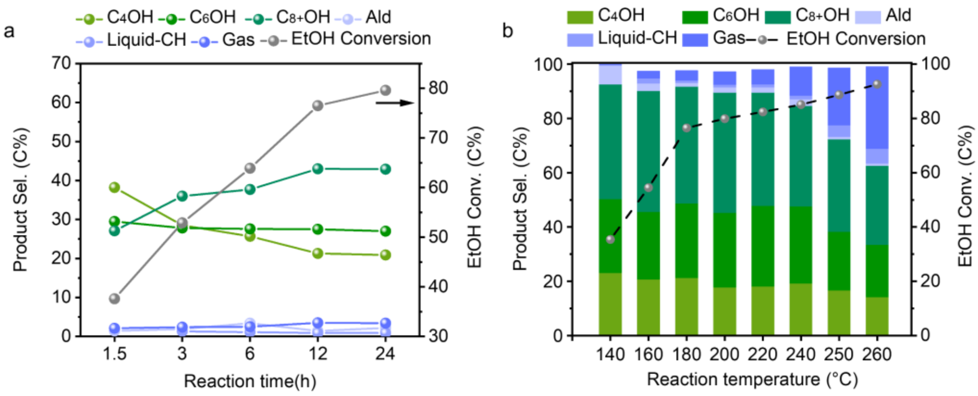


**Supplementary Fig. 13** | The performance of Ni@C-$S_{1/30}$ catalyst under different reaction conditions. (**a**) EtOH conversion and product selectivity for various reaction times at 180 °C. (**b**) EtOH conversion and product selectivity at various temperatures within 12 h. Reaction conditions: catalyst (0.3 g), NaOH (21.6 mmol), 50.0 wt% aqueous EtOH (108.0 mmol).

Supplementary Fig. 12a indicates that the EtOH conversion continues to increase with the reaction time within 12 h. Moreover, the $C_4OH$ and $C_6OH$ selectivity decreases along with the gradual growth of $C_{8+}OH$ selectivity, indicating that prolonging the reaction time can enhance the carbon-chain propagation. However, the EtOH conversion and product selectivity gradually reach steady values when the reaction time is further prolonging. Supplementary Fig. 12b indicates that raising the reaction temperature can significantly increase the ethanol conversion in the range of 140 °C to 180 °C. As the reaction temperature is over 180 °C, the severe side reaction of C−C bond cleavage is intensified, which limits the Guerbet condensation proceeding.

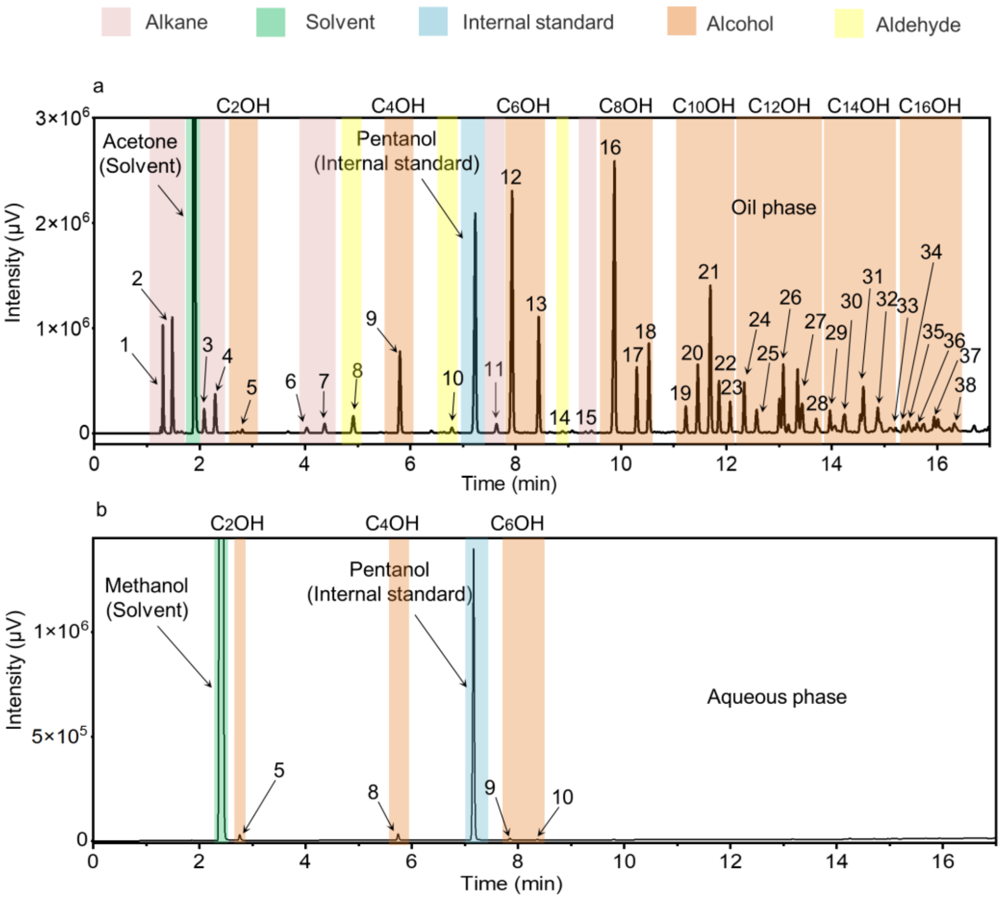


**Supplementary Fig. 14** | GC profiles of liquid products obtained from a two-stage intensification process reaction (180 °C for 6 h and then heating to 250 °C for another 6 h) over the Ni@C-$S_{1/30}$ catalyst.

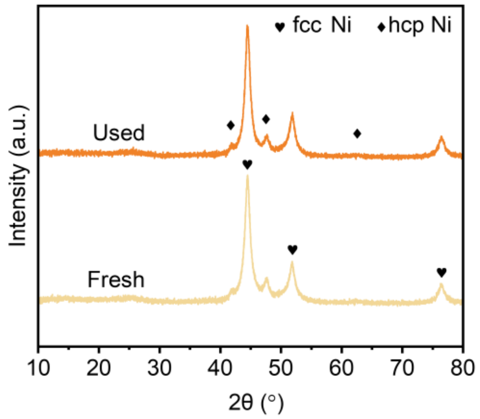


**Supplementary Fig. 15 |** XRD patterns of the Ni@C-$S_{1/30}$ catalyst before and after six reaction cycles.

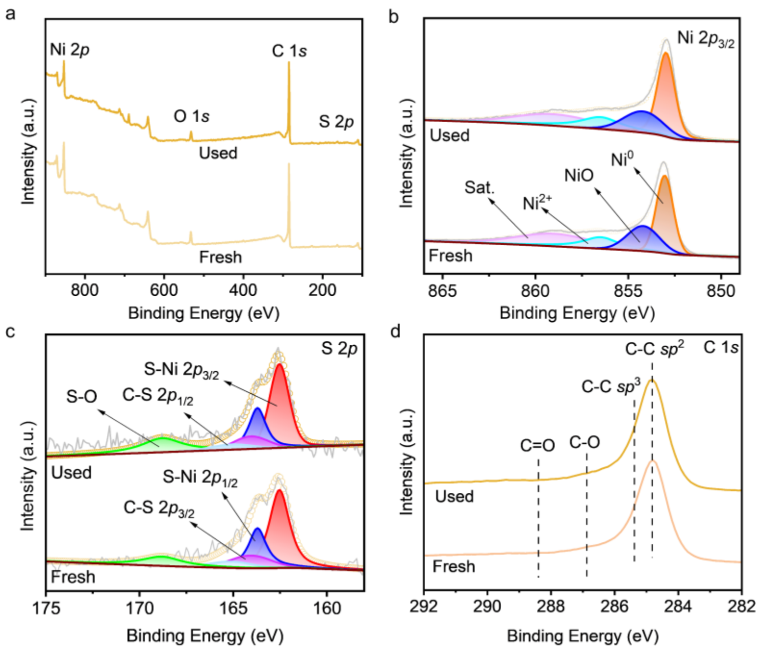


**Supplementary Fig. 16** | (**a**) XPS survey spectra. High-resolution (**b**) Ni 2$p_{3/2}$, (**c**) S 2$p$ and (**d**) C 1$s$ XPS spectra for the Ni@C-$S_{1/30}$ catalyst before and after six reaction cycles.

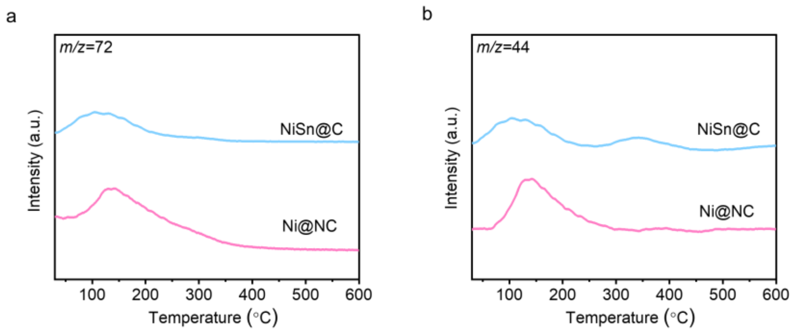


**Supplementary Fig. 17** | *n*-Butanal-TPD/MS profiles over the NiSn@C and Ni@NC catalysts: (**a**) Signals of *n*-butanal (*m/z* = 72) and (**b**) signals of 1-propane (*m/z* = 44).

As shown in Supplementary Fig. 16, the desorption peaks below 150 °C are attributed to the weak adsorption of *n*-butanal on the NiSn@C and Ni@NC catalysts. Only a weak peak in the region of 280–440 °C can be observed at *m/z* = 44 over the NiSn@C catalyst, and almost no dissociation peak can be observed over the Ni@NC catalyst, indicating the lack of strong adsorption sites for aldehydes on these catalysts.

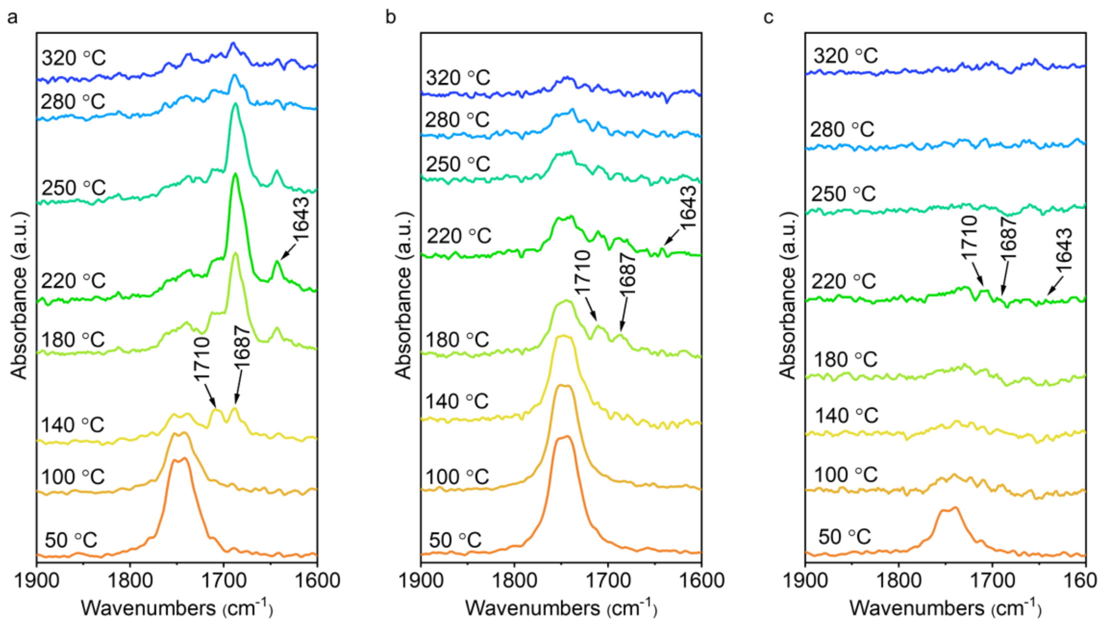


**Supplementary Fig. 18** | Temperature-dependent *in situ* DRIFTS spectra in the range of 1600–1900 $cm^{-1}$ for *n*-butanal adsorption on the (**a**) Ni@C-$S_0$, (**b**) Ni@C-$S_{1/30}$ and (**c**) Ni@C-$S_{1/25}$ catalysts.

The *in situ* DRIFTS spectra in the range of 1600–1900 $cm^{-1}$ were recorded to observe the generated 2-butenal molecules over Ni@C-$S_x$ catalysts. As shown in Supplementary Fig. 17, the bands at 1643 and 1687 $cm^{-1}$ can be assigned to the *v*(C=C) and *v*(C=O) of 2-butenal coordinated on the catalyst surface, respectively, while the band at 1710 $cm^{-1}$ is attributed to the *v*(C=O) of unabsorbed or physically absorbed 2-butenal molecule. Several changes occur in the relative peak intensities as the spectra evolve with increasing S/Ni ratio. Both bonds of 2-butenal decreased and the intensity of peaks centered at 1710 $cm^{-1}$ become more prominent, indicating that the dissociation of *n*-butanal is suppressed over S-doped Ni surface, which is in good agreement with the results of experiments.

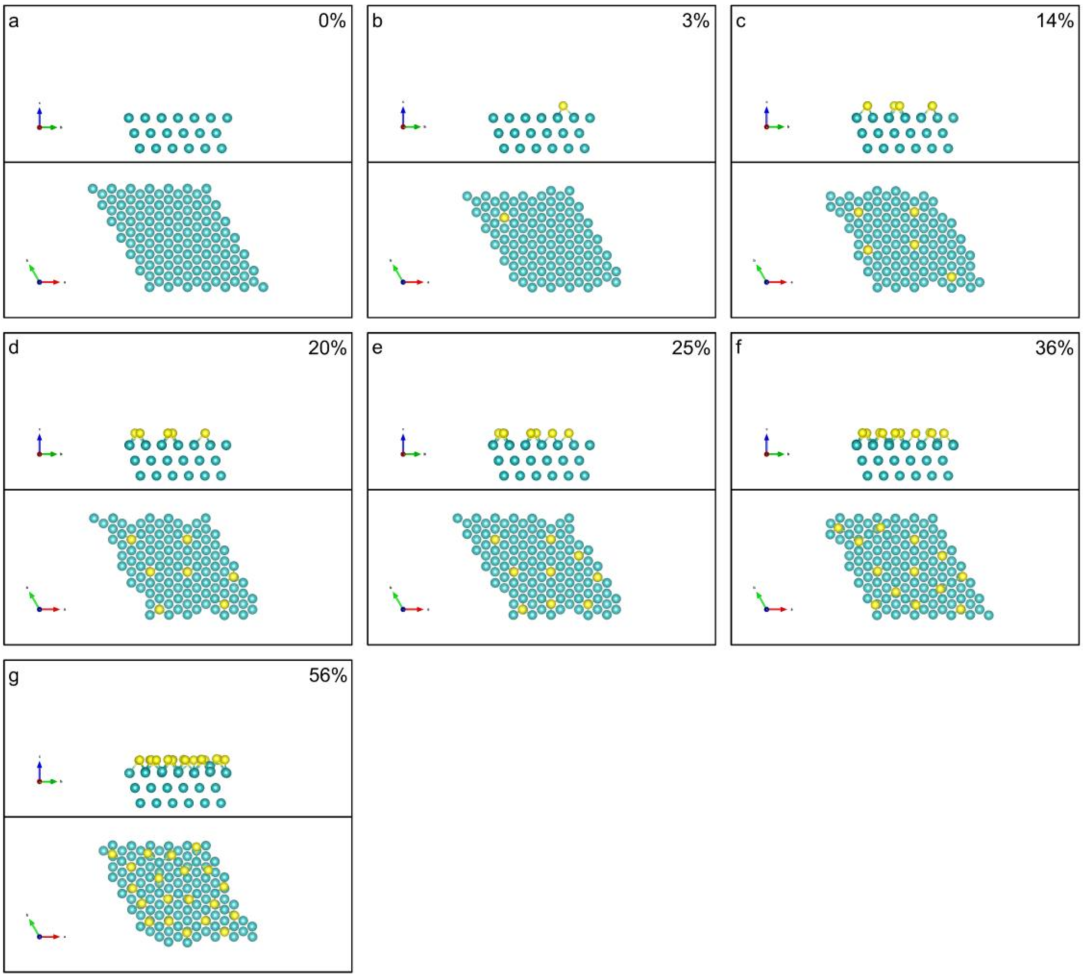


**Supplementary Fig. 19** | Catalyst models of Ni (111) with different sulfur coverage: (**a**) 0%, (**b**) 3%, (**c**) 14%, (**d**) 20%, (**e**) 25%, (**f**) 36%, (**g**) 56%.

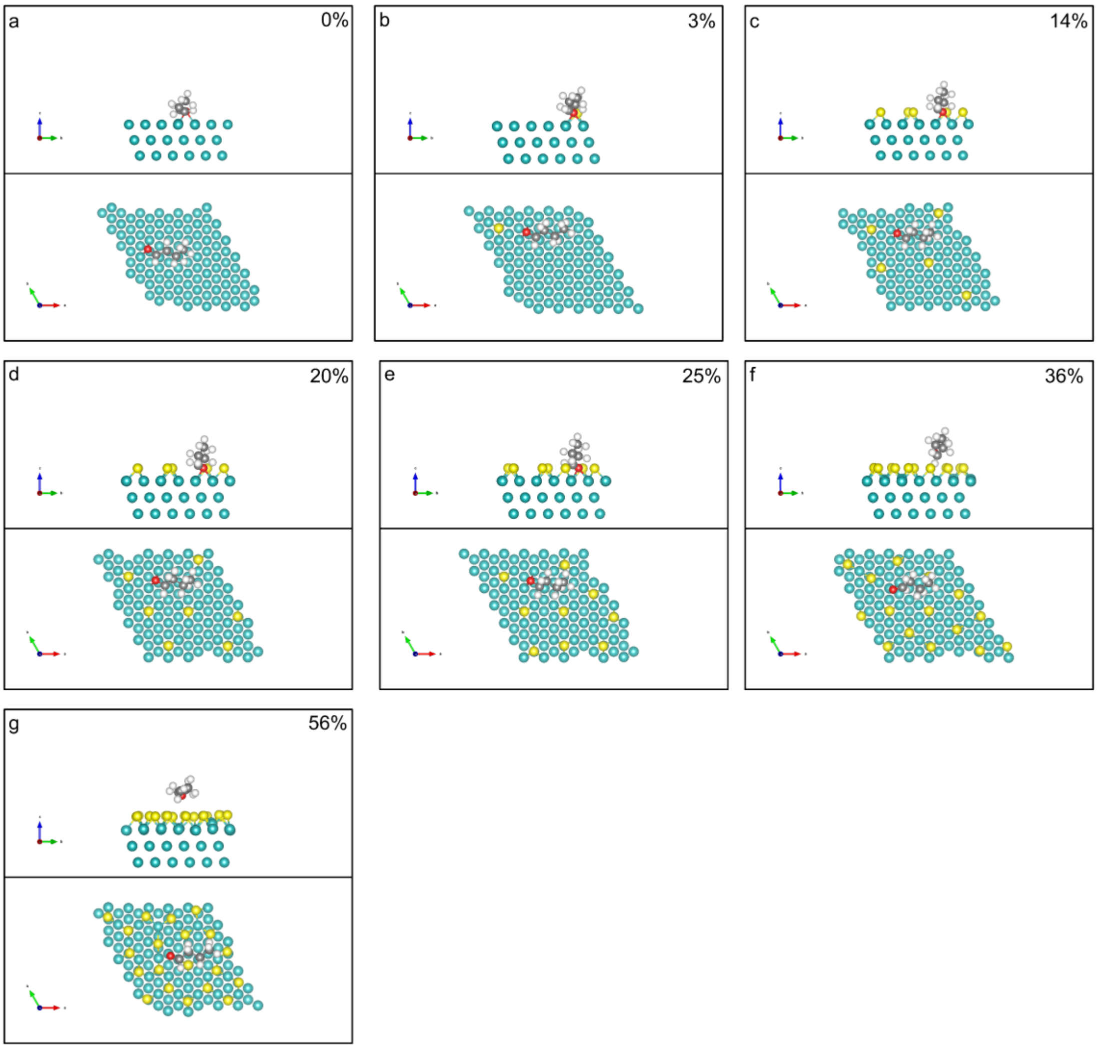


**Supplementary Fig. 20** | The stable adsorption configurations of *n*-butanal molecule on Ni (111) with different sulfur coverage: (**a**) 0%, (**b**) 3%, (**c**) 14%, (**d**) 20%, (**e**) 25%, (**f**) 36%, (**g**) 56%.

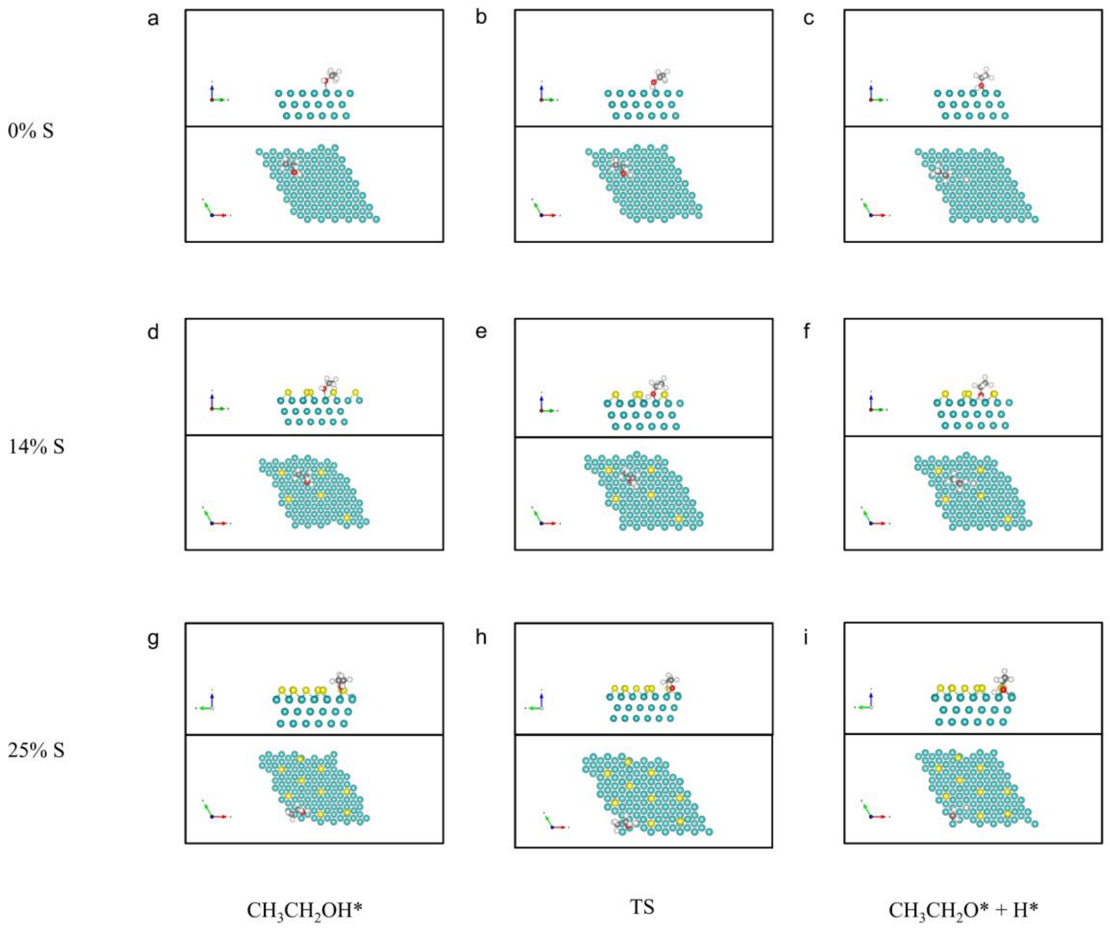


**Supplementary Fig. 21** | Configurations of $CH_3CH_2OH^*$ dehydrogenation to $CH_3CH_2O^*$ on Ni (111) with different sulfur coverage: (**a-c**) 0%, (**d-f**) 14%, (**g-i**) 25%.

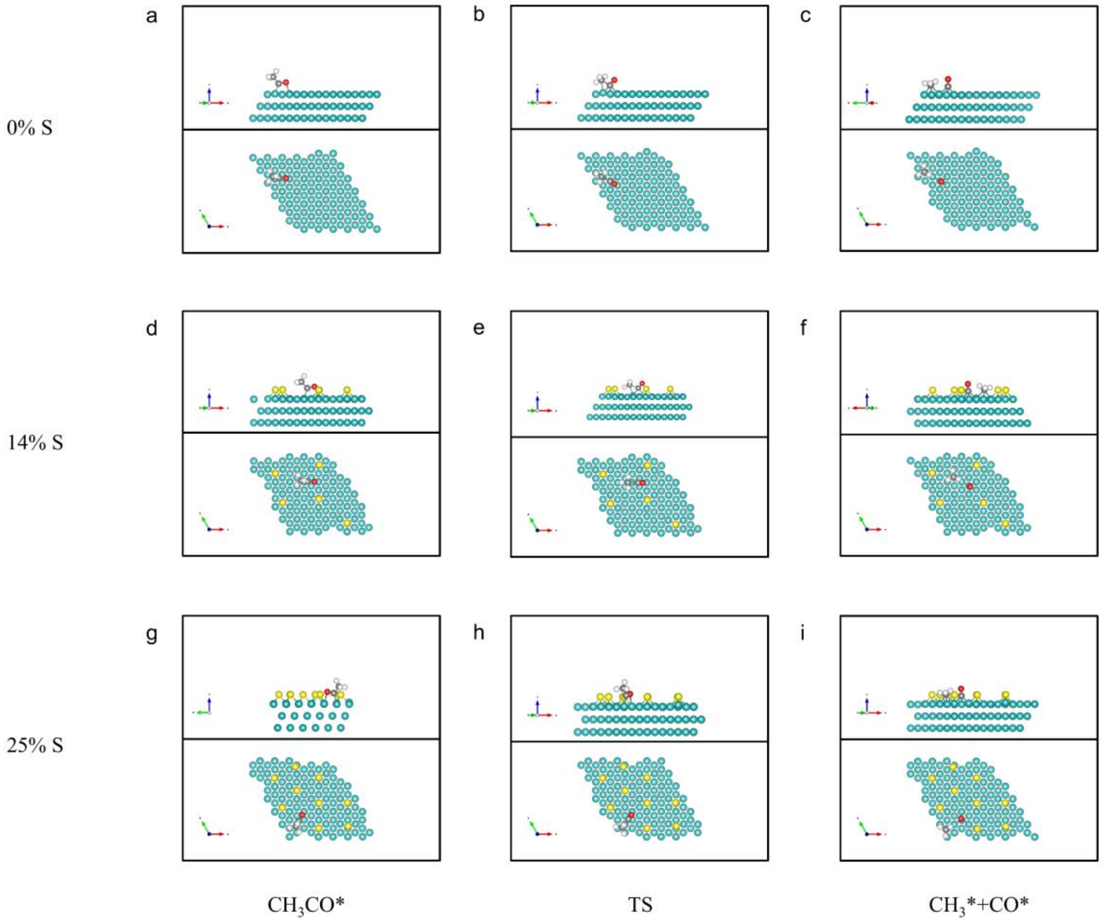


**Supplementary Fig. 22 |** Configurations of intermediate $CH_3CO$* decomposition to $CH_3$* and CO* on Ni (111) with different sulfur coverage: (**a-c**) 0%, (**d-f**) 14%, (**g-i**) 25%.

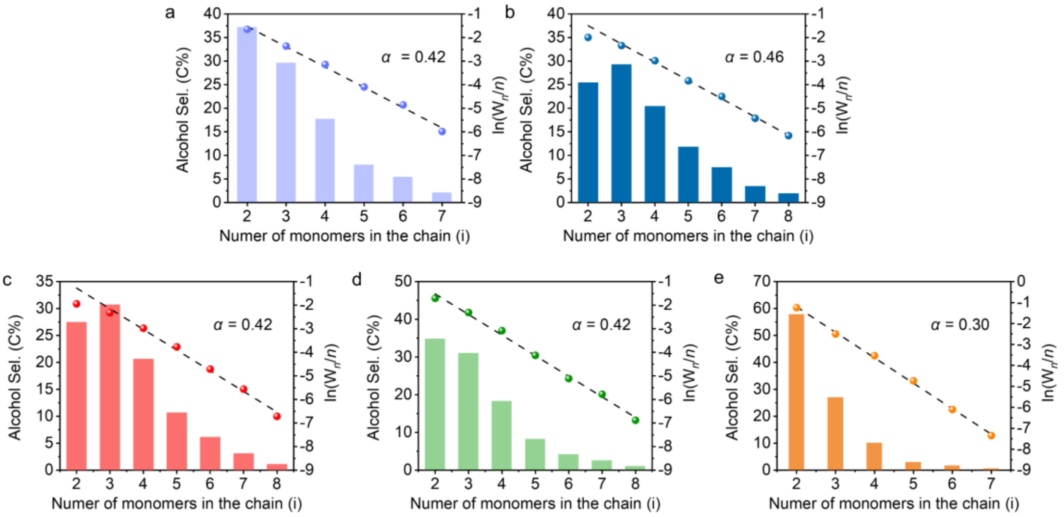


**Supplementary Fig. 23 |** Detailed alcohol distribution, step-growth plots and $\alpha$ values as a function of DMSO fraction in aqueous EtOH over the Ni@C-$S_0$ catalyst: (**a**) 0, (**b**) 0.006, (**c**) 0.009, (**d**) 0.012, (**e**) 0.024.

Dimethyl sulfoxide (DMSO) as a sulfur-containing specie was directly introduced to modify the Ni surface over the Ni@C-$S_0$ catalyst. As shown in Supplementary Fig. 22, the increase of $\alpha$ and the deviation of ln($W_n/n$) value for butanol can also be observed as the DMSO fraction increases from 0 to 0.009%. These results further suggest the essential role of sulfur on Ni surface in targeted production of LAS over Ni@C-$S_x$ catalyst.

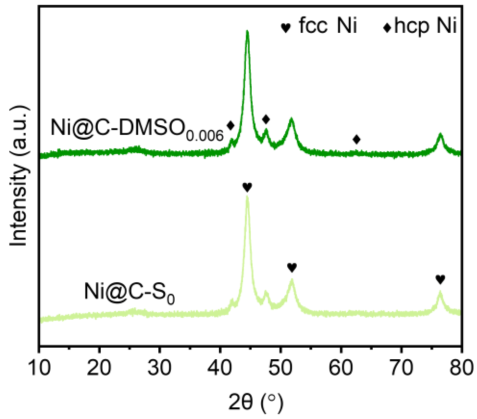


**Supplementary Fig. 24** | XRD patterns of the Ni@C-$S_0$ catalyst before and after reaction in aqueous EtOH solution with 0.006% DMSO fraction.

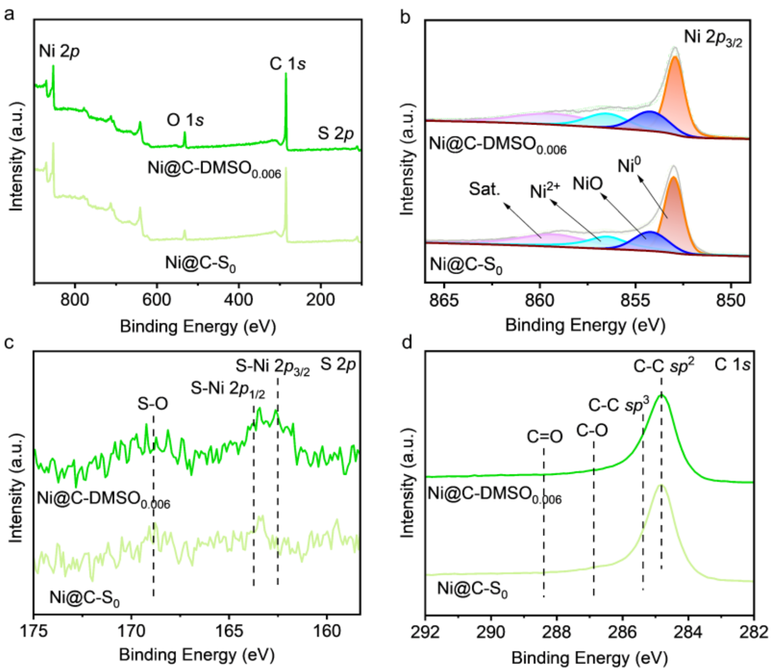


**Supplementary Fig. 25 |** (**a**) XPS survey spectra. High-resolution (**b**) Ni 2$p_{3/2}$, (**c**) S 2$p$ and (**d**) C 1$s$ XPS spectra for the Ni@C-$S_0$ catalyst before and after reaction in aqueous EtOH solution with 0.006% DMSO fraction.

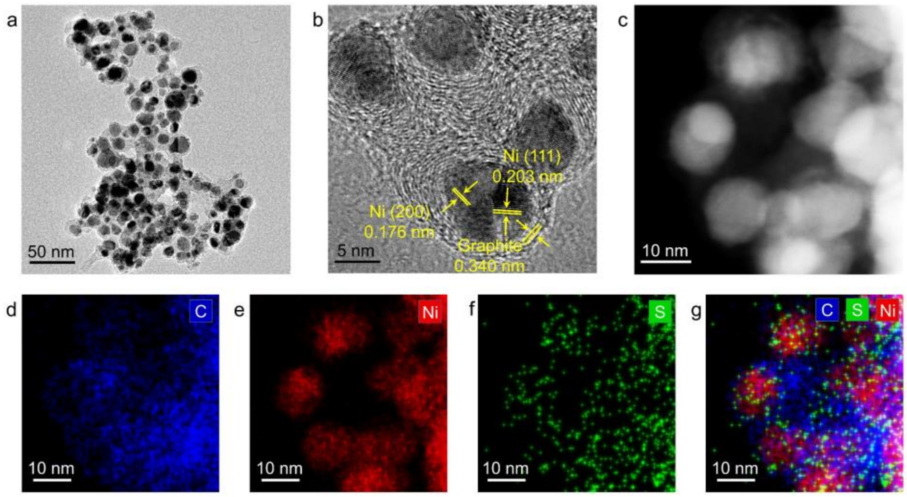


**Supplementary Fig. 26** | (**a**) TEM image, (**b**) HRTEM image, (**c**) HAADF-STEM image and (**d-g**) EDS elemental mappings of the Ni@C-$DMSO_{0.006}$ catalyst.

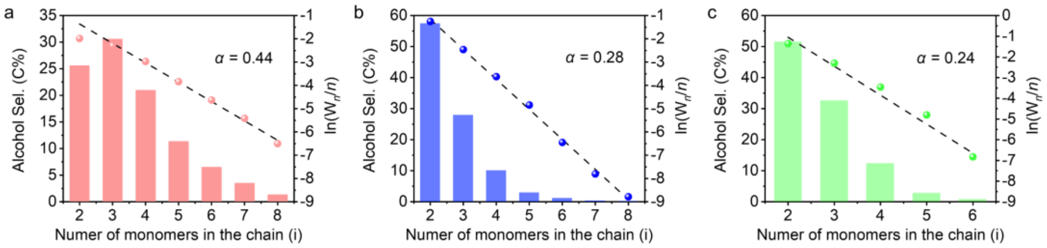


**Supplementary Fig. 27** | Detailed alcohol distribution, step-growth plots and $\alpha$ values over Ni@C-$S_{1/30}$-y catalysts prepared with other sulfur precursors: (**a**) $NiSO_4$, (**b**) LA and (**c**) SDS.

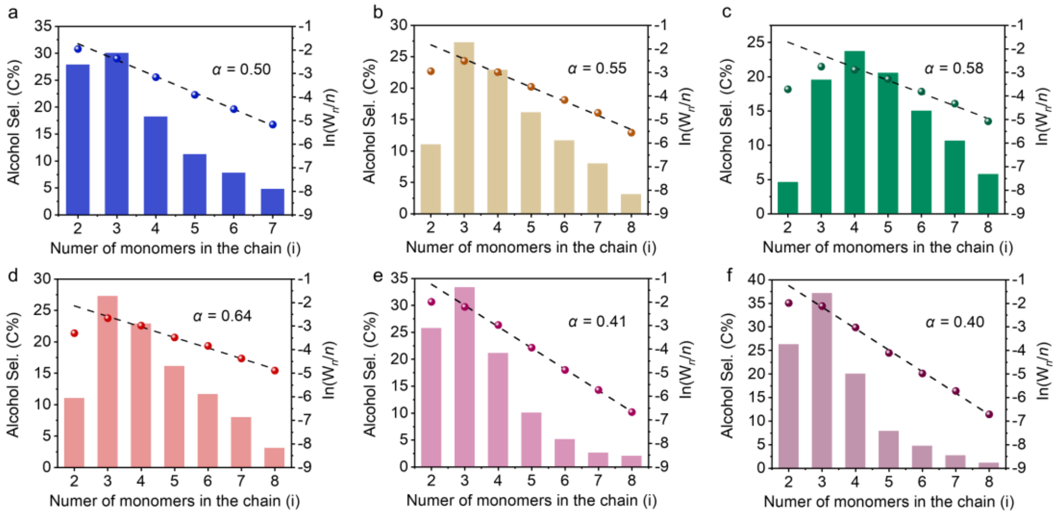


**Supplementary Fig. 28** | Detailed alcohol distribution, step-growth plots and $\alpha$ values over different catalysts under optimized conditions: (**a**) Ni@C-$S_0$, (**b**) Ni@C-$DMSO_{0.006}$, (**c**) Ni@C-$S_{1/30}$, (**d**) Ni@C-$S_{1/30}$-$NiSO_4$, (**e**) Ni@C-$S_{1/30}$-LA and (**f**) Ni@C-$S_{1/30}$-SDS. Reaction conditions: 180 °C for 6 h and then heating to 250 °C for 6 h, catalyst (0.3 g), NaOH (21.6 mmol), 50.0 wt% aqueous EtOH (108.0 mmol).

# 4. Supplementary Tables

**Supplementary Table 1 |** Chemical compositions and textural properties of typical Ni@C-$S_x$ catalysts.

| Catalyst | $C^a$ (wt%) | $S^a$ (wt%) | $Ni^b$ (wt%) | $Ni/S^c$ | $Ni/S^d$ | $S_{BET}{}^e$ ($m^2\cdot g^{-1}$) | Pore volume[e] ($cm^3\cdot g^{-1}$) | Pore diameter[e] (nm) |
|---|---|---|---|---|---|---|---|---|
| Ni@C-$S_0$ | 28.1 | – | 65.6 | – | – | 149.8 | 0.35 | 9.5 |
| Ni@C-$S_{1/30}$ | 22.6 | 1.3 | 67.4 | 28.4 | 5.7 | 131.5 | 0.39 | 11.6 |
| Ni@C-$S_{1/25}$ | 25.3 | 1.6 | 66.8 | 22.8 | 5.4 | 101.3 | 0.37 | 14.4 |

[a]S and C contents in the catalysts were determined by elemental analysis.

[b]Ni contents in the catalysts were determined by ICP–OES analysis.

[c]The Ni/S atomic ratios were calculated from elemental analysis and ICP–OES results.

[d]The Ni/S atomic ratios were calculated from XPS results.

[e]Surface areas were calculated using standard BET theory. Pore volumes and diameters of the samples were calculated using BJH theory.

**Supplementary Table 2 |** Catalytic performance of aqueous EtOH coupling over Ni@C-$S_x$ catalysts with different S/Ni molar ratios.[a]

| S/Ni molar ratio | Conversion (%) | Carbon balance (%) | Selectivity (C%) | | | | | | |
|---|---|---|---|---|---|---|---|---|---|
| | | | $C_4OH$ | $C_6OH$ | $C_{8+}OH$ | Ald | L-CH | Gas | Other |
| 0 | 51.8 | 86.2 | 29.2 | 23.2 | 26.0 | 2.4 | 1.1 | 16.0 | 2.1 |
| 1/60 | 66.2 | 87.6 | 25.4 | 27.1 | 34.0 | 2.9 | 1.5 | 7.2 | 1.9 |
| 1/30 | 76.5 | 84.8 | 21.3 | 27.5 | 43.0 | 1.4 | 0.9 | 3.5 | 2.4 |
| 1/25 | 62.5 | 85.7 | 44.1 | 29.3 | 17.7 | 4.0 | 1.3 | 1.3 | 2.3 |
| 1/20 | 29.0 | 90.7 | 72.5 | 15.3 | 4.8 | 7.0 | – | 0.4 | – |

[a]Reaction conditions: 180 °C, 12 h, catalyst (0.3 g), NaOH (21.6 mmol), 50.0 wt% aqueous EtOH (108.0 mmol). ($C_4OH$: *n*-butanol, $C_6OH$: *n*-hexanol and 2-ethyl-1-butanol, $C_{8+}OH$: $C_{8+}$ alcohols, Ald: aldehydes, L-CH: liquid hydrocarbon products, Gas: gaseous phase carbon products, Other: unidentified species).

**Supplementary Table 3** | EtOH conversion and product selectivity as a function of reaction time over the Ni@C-$S_{1/30}$ catalyst.[a]

| Reaction time (h) | Conversion (%) | Carbon balance (%) | Selectivity (C%) | | | | | | |
|---|---|---|---|---|---|---|---|---|---|
| | | | $C_4OH$ | $C_6OH$ | $C_{8+}OH$ | Ald | L-CH | Gas | Other |
| 1.5 | 37.6 | 86.2 | 38.2 | 29.5 | 27.1 | 1.4 | – | 2.1 | 1.7 |
| 3 | 52.9 | 84.0 | 28.5 | 27.8 | 36.0 | 1.9 | 1.3 | 2.4 | 2.1 |
| 6 | 63.9 | 83.8 | 25.7 | 27.6 | 37.7 | 3.4 | 1.1 | 2.5 | 2.0 |
| 12 | 76.5 | 84.8 | 21.3 | 27.5 | 43.0 | 1.4 | 0.9 | 3.5 | 2.4 |
| 24 | 79.6 | 82.1 | 20.9 | 27.0 | 42.9 | 2.2 | 0.9 | 3.4 | 2.7 |

[a]Reaction conditions: 180 °C, 12 h, catalyst (0.3 g), NaOH (21.6 mmol), 50.0 wt% aqueous EtOH (108.0 mmol).

**Supplementary Table 4** | Effect of reaction temperature on the catalytic performance of Ni@C-$S_{1/30}$ catalyst.[a]

| Reaction temperature (°C) | Conversion (%) | Carbon balance (%) | Selectivity (C%) | | | | | | |
|---|---|---|---|---|---|---|---|---|---|
| | | | $C_4OH$ | $C_6OH$ | $C_{8+}OH$ | Ald | L-CH | Gas | Other |
| 140 | 35.4 | 85.7 | 23.2 | 27.2 | 42.2 | 7.1 | - | 0.2 | 0.1 |
| 160 | 54.5 | 84.7 | 20.8 | 24.9 | 44.5 | 2.8 | 1.8 | 2.6 | 2.6 |
| 180 | 76.5 | 84.8 | 21.3 | 27.5 | 43.0 | 1.4 | 0.9 | 3.5 | 2.4 |
| 200 | 79.9 | 81.9 | 17.9 | 27.4 | 44.3 | 1.9 | 1.0 | 4.7 | 2.8 |
| 220 | 82.4 | 80.4 | 18.2 | 29.7 | 41.8 | 1.9 | 1.1 | 5.4 | 1.9 |
| 240 | 85.1 | 78.0 | 19.3 | 28.5 | 37.0 | 2.5 | 1.3 | 10.5 | 0.9 |
| 250 | 88.7 | 76.9 | 16.7 | 21.6 | 34.1 | 0.9 | 4.2 | 21.0 | 1.5 |
| 260 | 92.6 | 75.7 | 14.3 | 19.3 | 29.1 | 0.9 | 5.4 | 30.2 | 0.8 |

[a]Reaction conditions: 12 h, catalyst (0.3 g), NaOH (21.6 mmol), 50.0 wt% aqueous EtOH (108.0 mmol).

**Supplementary Table 5** | Effect of two-stage heating program with different second stage temperatures on the catalytic performance of Ni@C-$S_{1/30}$ catalyst.[a]

| Second-stage temperature (°C) | Conversion (%) | Carbon balance (%) | Selectivity (C%) | | | | | | |
|---|---|---|---|---|---|---|---|---|---|
| | | | $C_4OH$ | $C_6OH$ | $C_{8+}OH$ | Ald | L-CH | Gas | Other |
| 220 | 90.7 | 85.7 | 12.1 | 22.8 | 48.5 | 0.6 | 3.8 | 11.0 | 1.2 |
| 240 | 96.6 | 84.9 | 7.3 | 19.2 | 53.6 | 0.7 | 6.0 | 11.7 | 1.5 |
| 250 | 99.1 | 85.7 | 3.6 | 15.2 | 59.0 | 0.6 | 6.2 | 13.4 | 2.0 |
| 260 | 99.0 | 86.2 | 3.4 | 13.0 | 55.7 | 0.8 | 8.6 | 17.1 | 1.4 |
| 280 | 99.0 | 87.1 | 3.8 | 13.7 | 51 | 0.8 | 8.5 | 20.4 | 1.8 |

[a]Reaction conditions: at 180 °C and then at the second stage temperature for 6 h, catalyst (0.3 g), NaOH (21.6 mmol), 50.0 wt% aqueous EtOH (108.0 mmol).

**Supplementary Table 6 |** Catalytic performance comparison among the Ni@C-$S_{1/30}$ catalyst and other previously reported catalysts.

| Catalyst | T (°C) | Other reaction condition | EtOH conv. (C%) | LAS sel. (C%) | LAS yield. (C%) | Ref |
|---|---|---|---|---|---|---|
| **Ni@C-$S_{1/30}$-550** | **180-250** | **0.3 g catalyst, 5.0 g EtOH, 12 h** | **99.1** | **74.2** | **73.5** | **This work** |
| **Ni@C-$S_{1/30}$-550** | **180** | **0.3 g catalyst, 5.0 g EtOH, 12 h** | **76.5** | **70.5** | **53.9** | **This work** |
| BAP-0.25 Ni | 200 | 0.25 g catalyst, 0.5 mL EtOH, 24 h | 55.6 | 67.7 | 37.6 | 12 |
| Cu-HAP | 300 | WHSV = 2.0 $h^{-1}$ | 63.5 | 48.6 | 30.9 | 13 |
| HAP-W | 325 | WHSV = 0.7 $h^{-1}$ | 45.7 | 63.9 | 29.2 | 14 |
| $Ni_{20}Sn_1$@NC | 250 | 0.5g catalyst, 15 g EtOH, 24 h | 68.5 | 35.0 | 24.0 | 15 |
| NiSnH | 250 | 0.5 g catalyst, 15 g EtOH, 24 h | 46.6 | 49.2 | 22.9 | 16 |
| NiSn/MgAlO | 250 | 0.6 g catalyst, 10 g EtOH, 12 h | 66.9 | 33.7 | 22.5 | 17 |
| NiSn@C | 250 | 0.5 g catalyst, 15 g EtOH, 24 h | 46.9 | 47.7 | 22.4 | 1 |
| [Ir(OAc)$_3$]–L10 (1 : 10) | 150 | 0.01 g catalyst, 1g EtOH, 16 h | 52.0 | 30.7 | 16.0 | 18 |
| 2%Pd@UiO-66 | 250 | LHSV = 4 mL·$g^{-1}$·$h^{-1}$ | 49.9 | 27.9 | 13.9 | 19 |
| $Cu_{10}Ni_{10}$-PMO | 320 | 0.1 g catalyst, 3 mL EtOH, 6 h | 56.0 | 23.6 | 13.2 | 20 |
| 3Cu1Ce/AC | 250 | LHSV = 4 mL·$g^{-1}$·$h^{-1}$ | 46.2 | 20.6 | 9.5 | 21 |
| $FeNiO_x$ | 230 | 0.3 g catalyst, 30 mL EtOH, 24 h | 28.0 | 29.0 | 8.1 | 22 |
| NiMgAlO | 250 | WHSV = 3.2 $h^{-1}$ | 18.8 | 31.1 | 5.8 | 23 |

[a]The yields of LAS were calculated based on the following formula: LAS yield (C%) = Conversion ×Selectivity.

**Supplementary Table 7 |** Stability of the Ni@C-$S_{1/30}$ catalyst.[a]

| Run | Conversion (%) | Carbon balance (%) | Selectivity (C%) | | | | | | |
|---|---|---|---|---|---|---|---|---|---|
| | | | $C_4OH$ | $C_6OH$ | $C_{8+}OH$ | Ald | L-CH | Gas | Other |
| 1 | 99.1 | 85.7 | 3.6 | 15.2 | 59.0 | 0.6 | 6.2 | 13.4 | 2.0 |
| 2 | 99.1 | 85.2 | 4.8 | 17.1 | 57.5 | 0.7 | 6.3 | 12.3 | 1.3 |
| 3 | 98.6 | 83.6 | 6.5 | 19.8 | 54.5 | 0.7 | 7.0 | 10.8 | 0.7 |
| 4 | 97.5 | 84.8 | 7.4 | 19.7 | 55.4 | 0.8 | 4.9 | 10.6 | 1.2 |
| 5 | 97.1 | 83.7 | 7.6 | 21.7 | 54.8 | 0.6 | 4.3 | 10.0 | 1.0 |
| 6 | 95.7 | 82.0 | 7.0 | 20.6 | 59.0 | 0.7 | 3.0 | 9.1 | 0.6 |

[a]Reaction conditions: At 180 °C and then at 250 °C for 6 h respectively, catalyst (0.3 g), NaOH (21.6 mmol), 50.0 wt% aqueous EtOH (108.0 mmol).

**Supplementary Table 8** | Catalytic performance of aqueous EtOH coupling over typical Ni@C-$S_x$ catalysts with different S/Ni molar ratios.[a]

| Catalyst | Conversion (%) | Carbon balance (%) | Selectivity (C%) | | | | | | |
|---|---|---|---|---|---|---|---|---|---|
| | | | $C_4OH$ | $C_6OH$ | $C_{8+}OH$ | Ald | L-CH | Gas | Other |
| Ni@C-$S_0$ | 32.7 | 85.8 | 37.6 | 26.5 | 26.2 | – | – | 8.5 | 1.3 |
| Ni@C-$S_{1/30}$ | 37.6 | 86.2 | 38.2 | 29.5 | 27.1 | 1.4 | – | 2.1 | 1.7 |
| Ni@C-$S_{1/25}$ | 36.6 | 84.5 | 47.9 | 27.1 | 18.4 | 3.6 | – | 1.7 | 1.3 |

[a]Reaction conditions: 180 °C, 1.5 h, catalyst (0.3 g), NaOH (21.6 mmol), 50.0 wt% aqueous EtOH (108.0 mmol).

**Supplementary Table 9** | Catalytic performance of Ni@C-$S_x$ catalysts for 2-butenal hydrogenation.[a]

| Catalyst | Conversion (%) | Selectivity (C%) | | Carbon balance (%) |
|---|---|---|---|---|
| | | *n*-Butanal | *n*-Butanol | |
| Ni@C-$S_0$ | 98.9 | 15.4 | 84.6 | 92.2 |
| Ni@C-$S_{1/30}$ | 91.9 | 72.2 | 27.8 | 90.3 |
| Ni@C-$S_{1/25}$ | 90.2 | 97.8 | 2.2 | 86.6 |

[a]Reaction conditions: Catalyst (0.05 g), 50.0 wt% EtOH (40.0 mL), 2-butenal (14.0 mmol), 180 °C, 2 MPa $H_2$, 1 h.

**Supplementary Table 10** | Dehydrogenation and hydrogenation rate of Ni@C-$S_x$ catalysts.[a]

| Catalyst | EtOH dehydrogenation rate (mmol $g_{cat}^{-1}$ $h^{-1}$) | Hydrogenation rate (mmol $g_{cat}^{-1}$ $h^{-1}$) |
|---|---|---|
| Ni@C-$S_0$ | 157.0 | 1022.4 |
| Ni@C-$S_{1/30}$ | 180.5 | 657.7 |
| Ni@C-$S_{1/25}$ | 175.7 | 516.2 |

[a]Calculated from the results in Supplement Table S8 and Table S9.

**Supplementary Table 11** | Aldol condensation of *n*-butanal catalyzed by NaOH.[a]

| Catalyst | Conv. (%) | 2-ethyl-2-hexenal sel. (C %) | C−C formation rate (mmol $g_{cat}^{-1}$ $h^{-1}$) |
|---|---|---|---|
| NaOH | 100 | 100 | >2082 |

[a]Reaction conditions: NaOH: (0.1 g), 50.0 wt% EtOH (40.0 mL), *n*-butanal (69.4 mmol), room temperature, 10 min.

**Supplementary Table 12 |** Surface sulfur coverage and Ni active sites of typical Ni@C-$S_x$ catalysts[a].

| Catalyst | Sulfur coverage (%) | Ni active sites ($\mu mol \cdot g^{-1}$) |
|---|---|---|
| Ni@C-$S_0$ | 0.0 | 26.9 |
| Ni@C-$S_{1/30}$ | 13.6 | 15.9 |
| Ni@C-$S_{1/25}$ | 23.7 | 7.8 |

[a]Surface sulfur coverage and Ni active sites were determined by $N_2O$ titration.

**Supplementary Table 13** | Catalytic performance of aqueous EtOH coupling over Ni@C-DMSO$_z$ catalysts with different DMSO fractions in aqueous EtOH.[a]

| DMSO fraction (mol%) | Conversion (%) | Carbon balance (%) | Selectivity (C%) | | | | | | |
|---|---|---|---|---|---|---|---|---|---|
| | | | $C_4OH$ | $C_6OH$ | $C_{8+}OH$ | Ald | L-CH | Gas | Other |
| 0 | 51.8 | 86.2 | 29.2 | 23.2 | 26.0 | 2.4 | 1.1 | 16.0 | 2.1 |
| 0.006 | 76.9 | 86.0 | 22.4 | 25.8 | 39.8 | 1.1 | 1.3 | 8.0 | 1.6 |
| 0.009 | 70.3 | 87.4 | 24.1 | 27.0 | 36.7 | 3.4 | 0.9 | 5.5 | 2.4 |
| 0.012 | 66.1 | 83.7 | 31.8 | 28.3 | 31.2 | 2.6 | 1.1 | 2.6 | 2.4 |
| 0.024 | 46.6 | 88.2 | 52.3 | 24.4 | 13.7 | 5.2 | 1.6 | 2.4 | 0.4 |

[a]Reaction conditions: 180 °C, 12 h, catalyst (0.3 g), NaOH (21.6 mmol), 50.0 wt% aqueous EtOH (108.0 mmol).

**Supplementary Table 14 |** Catalytic performance of aqueous EtOH coupling over unmodified Ni@C-$S_0$ and Ni@C-$S_{1/30}$-y catalysts prepared with different sulfur precursors.[a]

| Sulfur precursor | Conversion (%) | Carbon balance (%) | Selectivity (C%) | | | | | | |
|---|---|---|---|---|---|---|---|---|---|
| | | | $C_4OH$ | $C_6OH$ | $C_{8+}OH$ | Ald | L-CH | Gas | Other |
| – | 51.8 | 86.2 | 29.2 | 23.2 | 26.0 | 2.4 | 1.1 | 16.0 | 2.1 |
| L-Cys | 76.5 | 84.8 | 21.3 | 27.5 | 43.0 | 1.4 | 0.9 | 3.5 | 2.4 |
| $NiSO_4$ | 71.9 | 83.1 | 23.8 | 28.5 | 40.7 | 1.0 | 1.1 | 2.4 | 2.5 |
| LA | 60.8 | 86.2 | 52.6 | 25.5 | 13.4 | 3.8 | 1.6 | 1.1 | 2.0 |
| SDS | 60.5 | 80.9 | 47.5 | 30.0 | 14.7 | 0.8 | 1.2 | 3.6 | 2.1 |

[a]Reaction conditions: 180 °C, 12 h, catalyst (0.3 g), NaOH (21.6 mmol), 50.0 wt% aqueous EtOH (108.0 mmol).

**Supplementary Table 15 |** The catalytic performance of typical catalysts under the optimized two-stage intensification process.[a]

| Catalyst | Conversion (%) | Carbon balance (%) | Doping process | Selectivity (C%) | | | | | | |
|---|---|---|---|---|---|---|---|---|---|---|
| | | | | $C_4OH$ | $C_6OH$ | $C_{8+}OH$ | Ald | L-CH | Gas | Other |
| Ni@C | 84.0 | 86.4 | – | 9.1 | 9.8 | 13.7 | 0.6 | 2.2 | 64.5 | 0.1 |
| Ni@C-$DMSO_{0.006}$ | 97.9 | 84.7 | Reaction | 7.2 | 17.9 | 41.0 | 1.5 | 9.8 | 21.1 | 1.5 |
| Ni@C-$S_{1/30}$ | 99.1 | 85.7 | Catalyst preparation | 3.6 | 15.2 | 59.0 | 0.6 | 6.2 | 13.4 | 2.0 |
| Ni@C-$S_{1/30}$-$NiSO_4$ | 94.6 | 86.3 | Catalyst preparation | 5.6 | 17.4 | 56.7 | 0.5 | 7.0 | 10.1 | 2.7 |
| Ni@C-$S_{1/30}$-LA | 82.1 | 83.5 | Catalyst preparation | 22.0 | 28.4 | 34.2 | 1.9 | 1.5 | 10.7 | 1.3 |
| Ni@C-$S_{1/30}$-SDS | 83.8 | 87.4 | Catalyst preparation | 21.5 | 30.4 | 29.9 | 2.6 | 1.2 | 12.4 | 2.0 |

[a]Reaction conditions: At 180 °C and then at 250 °C for 6 h respectively, catalyst (0.3 g), NaOH (21.6 mmol), 50.0 wt% aqueous EtOH (108.0 mmol).

## 5. Reference


1. Liu, W. *et al.* One-pot synthesis of high-carbon bio-alcohols from aqueous ethanol upgrading over water-tolerance NiSn@C catalyst. *Energy Convers. Manag.* **249**, 114822 (2021).
2. Gu, J. *et al.* N-rich doping strategy for constructing Ni@NC catalysts to boost aqueous ethanol coupling towards higher alcohols by inhibiting $C_1$ byproducts. *Chem. Eng. J.* **453**, 139583 (2023).
3. Chuang, S.S.C. & Pien, S.I. Enhancement of ethylene hydroformylation over $Ni/SiO_2$ through sulfur promotion. *Catal. Letters.* **6**, 389-393 (1990).
4. Greiner, G. & Menzel, D. Promotion and inhibition of $Ni(CO)_4$ formation on Ni(100): A kinetic investigation coupled with ESCA measurements. *J. Catal.* **77**, 382-396 (1982).
5. Greiner, G. & Menzel, D. Absence of magnetic field influences on the rate of $Ni(CO)_4$ formation on Ni(100) under various surface conditions. *Surf. Sci. Lett.* **109**, L510-L512 (1981).
6. Chuang, S.S.C., Pien, S.I. & Sze, C. Ethylene addition to CO hydrogenation over Sulfided Ni, Rh, and Ru. *J. Catal.* **126**, 187-191 (1990).
7. García-Diéguez, M., Finocchio, E., Larrubia, M.Á., Alemany, L.J. & Busca, G. Characterization of alumina-supported Pt, Ni and PtNi alloy catalysts for the dry reforming of methane. *J. Catal.* **274**, 11-20 (2010).
8. Xu, M. *et al.* Boosting CO hydrogenation towards $C_{2+}$ hydrocarbons over interfacial $TiO_{2-x}/Ni$ catalysts. *Nat. Commun.* **13**, 6720 (2022).
9. Yates, J.T., Jr. & Garland, C.W. Infrared study of carbon monoxide chemisorbed on nickel and on mercury-poisoned nickel surfaces[1]. *J. Phys. Chem.* **65**, 617-624 (1961).
10. Kubelkova´, L., Nova´kova´, J., Jaeger, N.I. & Schulz-Ekloff, G. Characterization of nickel species at $Ni/\gamma\text{-}Al_2O_3$ and Ni/faujasite catalysts by carbon monoxide adsorption. *Appl. Catal. A-Gen.* **95**, 87-101 (1993).
11. Meunier, F.C. In situ formation of $Ni(CO)_4$ contaminant during IR analyses using a metal-containing reaction cell. *Catal. Sci. Technol.* **12**, 7433-7438 (2022).
12. Xue, M., Yang, B., Xia, C. & Zhu, G. Upgrading Ethanol to Higher Alcohols via Biomass-Derived Ni/Bio-Apatite. *ACS Sustain. Chem. Eng.* **10**, 3466-3476 (2022).
13. Zhou, B.C. *et al.* Enhancing ethanol coupling to produce higher alcohols by tuning $H_2$ partial pressure over a Copper-hydroxyapatite catalyst. *ACS Catal.* **12**, 12045-12054 (2022).
14. Wang, Q.-N. *et al.* Hydroxyapatite nanowires rich in [Ca–O–P] sites for ethanol direct coupling showing high $C_{6–12}$ alcohol yield. *Chem. Commun.* **55**, 10420-10423 (2019).
15. Fei, X. *et al.* Aqueous phase catalytic conversion of ethanol to higher alcohols over NiSn bimetallic catalysts encapsulated in nitrogen-doped biorefinery lignin-based carbon. *Ind. Eng. Chem. Res.* **60**, 17959-17969 (2021).
16. Zhang, Q., Liu, W., Chen, B., Qiu, S. & Wang, T. Upgrading of aqueous ethanol to fuel grade higher alcohols over dandelion-like Ni-Sn catalyst. *Energy Convers. Manag.* **216**, 112914 (2020).
17. Wu, X. *et al.* Upgrading of aqueous bioethanol to higher alcohols over NiSn/MgAlO catalyst. *ACS Sustain. Chem. Eng.* **9**, 11269-11279 (2021).
18. Xu, G. *et al.* Direct self-condensation of bio-alcohols in the aqueous phase. *Green Chem.* **16**, 3971-3977 (2014).
19. Jiang, D. *et al.* Multifunctional Pd@UiO-66 Catalysts for Continuous Catalytic Upgrading of

Ethanol to n-Butanol. *ACS Catal.* **8**, 11973-11978 (2018).
20. Sun, Z. *et al.* Efficient Catalytic Conversion of Ethanol to 1-Butanol via the Guerbet Reaction over Copper- and Nickel-Doped Porous. *ACS Sustain. Chem. Eng.* **5**, 1738-1746 (2017).
21. Jiang, D., Wu, X., Mao, J., Ni, J. & Li, X. Continuous catalytic upgrading of ethanol to n-butanol over Cu–$CeO_2$/AC catalysts. *Chem. Commun.* **52**, 13749-13752 (2016).
22. Pang, J. *et al.* Catalytic upgrading of ethanol to butanol over a binary catalytic system of $FeNiO_x$ and LiOH. *Chinese J. Catal.* **41**, 672-678 (2020).
23. Pang, J. *et al.* Upgrading ethanol to n-butanol over highly dispersed Ni–MgAlO catalysts. *J. Catal.* **344**, 184-193 (2016).
